# Unlocking the complications of chronic concussion in rugby players: A traumatic tale of cerebral injury

**DOI:** 10.1113/EP091352

**Published:** 2023-08-08

**Authors:** John D. Akins, R. Matthew Brothers

**Affiliations:** ^1^ Institute for Exercise and Environmental Medicine at Texas Health Presbyterian Hospital Dallas Dallas TX USA; ^2^ The University of Texas Southwestern Medical Center Dallas TX USA; ^3^ The University of Texas at Arlington Arlington TX USA

**Keywords:** brain blood flow, concussion, neurocognitive function

1

As a vital organ controlling numerous bodily processes, the brain remains heavily studied for its role in blood pressure regulation, ventilation, motor processing and hunger among countless others. However, repeated trauma to the brain may result in irreversible damage that interferes with these vital processes. Such trauma may occur in daily life, though recent attention has focused on individuals participating in contact sports. Indeed, for nearly a century, the effects of subconcussive and concussive impacts/injuries to the head have been described in individuals across various sports including boxing, rugby, American football, ice hockey and martial arts (Gallo et al., 2020). Research has attempted to better understand the underlying physiological (e.g., autonomic, vascular), neurocognitive and psychiatric changes that accompany these head impacts/injuries (Ellis et al., 2016; Gallo et al., 2020). However, few, if any, studies to date have linked the cascade of events, including biomolecular pathways, vascular dysfunction and neurocognitive impairment, secondary to concussive injuries and how these factors change following a lifetime of exposure.

In the current issue of *Experimental Physiology*, Owens et al. (2023) present data from a cohesive study aimed at assessing the connections between biomolecular, cerebrovascular and neurocognitive outcomes in retired rugby union players with an established medical history of concussion. This study hypothesized that these players with a history of concussion, compared to otherwise healthy controls, would exhibit lower systemic bioactive nitric oxide (NO), which would be accompanied by impaired cerebrovascular and neurocognitive function. Therefore, Owens et al. (2023) studied retired rugby union players and matched controls during two visits. Visit 1 included a medical exam, dietary intake assessments and an exercise stress test. Visit 2 measured neurocognitive function (Montreal Cognitive Assessment), concussion history (Sports Concussion Assessment Tool), NO metabolites (e.g., nitrite, *S*‐nitrosothiols), neurovascular unit integrity (e.g., glial fibrillary acidic protein, neurofilament light‐chain), cerebral oxygen delivery ($CD_{O_{2}}$)), and cerebrovascular function assessed as cerebrovascular reactivity to hyper‐ and hypocapnia (i.e., changes in middle cerebral artery blood velocity (MCA_v_) per mmHg change in end‐tidal CO_2_).

As would be expected with chronic exposure to contact events, Owens et al. (2023) found that the players with a previous history of at least one concussion had persistent neurological symptoms. These symptoms included headache, worsened memory, and feelings of anxiety. Interestingly, the players also had a reduced dietary intake of carbohydrates, protein and several minerals. Moving to the biomolecular pathways, the players with a concussion history had lower basal bioactive NO, though no differences in markers of neurovascular unit integrity. Cerebrovascular function was also found to be impaired across a variety of conditions. At rest and during exposure to hypercapnia, absolute MCA_v_ and $CD_{O_{2}}$ were lower in players with concomitantly greater cerebrovascular resistance index and total peripheral resistance. These impairments in MCA_v_ and $CD_{O_{2}}$ similarly persisted during hypocapnia. Interestingly, once normalized as a relative change, cerebrovascular reactivity was not impaired in the players in response to either hyper‐ or hypocapnia nor was it different across the full reactivity range. The impairments in absolute cerebrovascular function may have some relation to the reduced bioactive NO as the authors found a moderate correlation between bioactive NO and the hypercapnic cerebrovascular reactivity. Finally, the players exhibited a greater presence of mild cognitive impairment, with a particular presentation of selective impairment in executive function and fine motor coordination of the non‐dominant hand. Overall, the authors found that retired rugby union players with a history of concussion were defined by the presence of lower systemic bioactive NO and persistent neurological symptoms, which were accompanied by cognitive impairment and cerebrovascular dysfunction at rest and in response to hyper‐/hypocapnia.

The current findings by Owens et al. (2023) naturally lead to questions related to the impacts of acute concussion on this biomolecular–cerebrovascular–neurocognitive cascade and where interventions may occur to help improve long‐term care in athletes exposed to repetitive contact activities. Indeed, data suggest that in the 7 days following a concussion in young athletes, dynamic changes in cerebral blood flow and microstructure seem to occur, though with a large degree of heterogeneity (Churchill et al., 2017). Accordingly, the repeated subconcussive and concussive head impacts/injuries experienced in contact sports may progressively alter cerebrovascular function and structure. The work presented by Owens et al. (2023) may also have a broader impact as annual traumatic brain injury (TBI)‐related emergency department visits number in the millions, with many TBI‐related hospitalizations and deaths in the general population (Capizzi et al., 2020). Therefore, understanding the underlying physiological mechanisms and the overall detrimental effect of repeated subconcussive head impacts or concussions are crucial for the proper acute and chronic treatment of concussion for improved long‐term outcomes in both athletes and non‐athletes. Regarding the current study, the authors should be commended for their advancement of our integrative understanding of how cerebral biomolecular pathways change in retired athletes with concussion history and how this may alter cerebrovascular and cognitive function.

## AUTHOR CONTRIBUTIONS

Both authors approved the final version of the manuscript and agree to be accountable for all aspects of the work in ensuring that questions related to the accuracy or integrity of any part of the work are appropriately investigated and resolved. Both persons designated as authors qualify for authorship, and all those who qualify for authorship are listed.

### CONFLICT OF INTEREST

The authors have no competing interests.

### FUNDING INFORMATION

There was no funding provided for this viewpoint article.
